# Molecular mechanisms of glucocorticoids on skeleton and bone regeneration after fracture

**DOI:** 10.1530/JME-18-0024

**Published:** 2018-03-27

**Authors:** Yasmine Hachemi, Anna E Rapp, Ann-Kristin Picke, Gilbert Weidinger, Anita Ignatius, Jan Tuckermann

**Affiliations:** 1 Institute of Comparative Molecular EndocrinologyUlm University, Ulm, Germany; 2 Institute of Orthopaedic Research and BiomechanicsUlm University Medical Centre, Ulm, Germany; 3 Institute of Biochemistry and Molecular BiologyUlm University, Ulm, Germany

**Keywords:** glucocorticoid receptor, osteoblast, osteoclast, bone regeneration, fracture healing

## Abstract

Glucocorticoid hormones (GCs) have profound effects on bone metabolism. Via their nuclear hormone receptor – the GR – they act locally within bone cells and modulate their proliferation, differentiation, and cell death. Consequently, high glucocorticoid levels – as present during steroid therapy or stress – impair bone growth and integrity, leading to retarded growth and glucocorticoid-induced osteoporosis, respectively. Because of their profound impact on the immune system and bone cell differentiation, GCs also affect bone regeneration and fracture healing. The use of conditional-mutant mouse strains in recent research provided insights into the cell-type-specific actions of the GR. However, despite recent advances in system biology approaches addressing GR genomics in general, little is still known about the molecular mechanisms of GCs and GR in bone cells. Here, we review the most recent findings on the molecular mechanisms of the GR in general and the known cell-type-specific actions of the GR in mesenchymal cells and their derivatives as well as in osteoclasts during bone homeostasis, GC excess, bone regeneration and fracture healing.

## Introduction

Glucocorticoid hormones (GCs) are major stress hormones released by a hierarchical hormonal axis. The circadian rhythm and psychological and physiological stress trigger the hypothalamus to release corticotropin-releasing factor (CRF), which in turn acts on the pituitary, stimulating its release of adrenocorticotropic hormone (ACTH). ACTH acts on the zona fasciculata of the adrenal cortex for the release of GCs, which belong to the steroid class of hormones. Cortisol as the major GC in humans and corticosterone, the major GC in rodents, act on virtually all cells in the body via the GC receptor (GR) (Baschant *et al*. 2012) and to a lesser extent through the mineralocorticoid receptor (MR), which displays a restricted expression pattern. GCs by acting on the brain and metabolic organs, including the liver, fat and muscle, contribute substantially to energy metabolism and tissue integrity by affecting cellular proliferation, differentiation, autophagy and apoptosis (Baschant & Tuckermann 2010). Consequently, energy is mobilised to ensure a fight-or-flight response. GCs have a substantial impact on the immune system. They belong to the most potent anti-inflammatory agents and are thus in widespread medical use to treat acute and chronic inflammation as well as pain. In addition, they are components of certain cancer therapies. High GC doses resulting from corticosteroid or from the stress response, induce a number of side effects, including strong effects on the musculoskeletal system. This leads to growth suppression in children and to GC-induced osteoporosis, which elevates the risk for bone fracture (Van Staa *et al*. 2000). Moreover, because distinct cells of the immune system and skeletal cells are involved in fracture healing, GCs modulate this response and disturbances might contribute to impaired healing responses.

Here, we review the recent mechanistic evidence of how GCs act via the GR and thus affect skeletal cells, and how this translates to alterations in bone mass and modification of fracture healing.

## General mechanisms of GC and GR action

Both endogenous and synthetic GCs exert their effects via nuclear receptors such as the GR (NR3C1) and the closely related mineralocorticoid receptor (MR) (NR3C2); both are ligand-induced transcription factors (De Bosscher & Haegeman 2009, Joëls & de Kloet 2017, van Weert & Meijer 2017). Only in certain MR-expressing tissues, in which GCs are not metabolically inactivated, the MR serves as a high-affinity receptor to mediate responses of low GC concentration. Due to the ubiquitous expression of the GR, the majority of GC effects are mediated by this receptor. In the absence of ligands, the GR is retained in the cytoplasm, because it is sequestered in a multiprotein complex that includes immunophilins and heat shock/chaperone proteins. Upon entering the cell, the bioavailability and activity of the GCs are controlled by the enzymes 11β-hydroxysteroid dehydrogenase (11β-HSD1) and 11β-HSD2, which act in an opposing manner, and regulate the relative levels of cortisone and cortisol (reviewed in Hartmann *et al*. 2016). Hormone binding induces a conformational change of the GR, allowing its translocation into the nucleus (Hartmann *et al*. 2016).

The ligand-bound GR can dimerize and bind directly to specific palindromic sequences in the genome (GC responsive elements (GRE) or glucocorticoid receptor-binding sites (GBS)) and in the vicinity to binding sites of tissue-specific transcription factors (reviewed in Hartmann *et al*. 2016). The bound GR molecules recruit co-regulator proteins and chromatin-remodelling complexes to increase or repress gene transcription (De Bosscher & Haegeman 2009, Surjit *et al*. 2011).

In addition to its action as a dimeric molecule, monomeric GR can bind directly to DNA (Schiller *et al*. 2014, Lim *et al*. 2015, Weikum *et al*. 2017) or tether to DNA-bound transcription factors involved in inflammation. Interaction with for instance nuclear factor kappa B (NF-κB), activator protein 1 (AP-1) and interferon response factor 3 (IRF3), leads to repression of gene expression (De Bosscher & Haegeman 2009). Previously, it was considered that monomer-dependent tethering of the GR was the main mechanism of the anti-inflammatory actions of GCs (De Bosscher & Haegeman 2009). This view was challenged by numerous studies using mice with a point mutation in one of the dimerization interfaces of the GR, GR^dim^ mice, demonstrating that GR dimer-dependent gene expression is indispensable for most anti-inflammatory effects (reviewed in Hübner *et al*. 2015). This is remarkable, because a point mutation in one of the GR dimerization domains does not entirely abrogate GR dimerization under *in vitro* conditions (Presman *et al*. 2014). However, genome-wide *in vivo* studies demonstrated an absence of binding to classical GR-binding sites (Lim *et al*. 2015). In addition to tethering, the GR can bind to a GRE half-site located within AP-1 response-element motifs. This direct interaction is important for transcriptional repression, and monomeric GR appears to be favoured at these sites (Weikum *et al*. 2017).

In addition to transcriptional activity, GCs can exert nongenomic effects. These effects are rapid and are only observed following high-dose GC treatment (Stahn & Buttgereit 2008). Under such conditions, GCs are considered to interact with plasma and mitochondrial membranes and affect their physicochemical properties, thereby altering their function. GCs can also bind to the cytosolic GR (cGR) and a membrane-bound GR (mGR) (Stahn & Buttgereit 2008), and then modulate mitogen-activated protein (MAP) kinase activity, leading to the regulation of other non-GR signalling pathways (reviewed in Hartmann *et al*. 2016). The impact of the nongenomic GR actions in bone cells and towards the contribution to GC effects on bone remains elusive.

Taken together, multiple molecular mechanisms are exerted by the GR to mediate GC effects. These include genomic effects by the induction and repression of gene expression in a cell-type-specific manner and nongenomic rapid effects in particular at high GC doses.

## GC action in bone – lessons from mouse models

In a hallmark study, Weinstein and colleagues (Weinstein *et al*. 1998) described a model of prednisolone slow release pellets leading to bone mineral density changes in Swiss Webster mice. Since then, the mouse became a popular model in preclinical GC-induced osteoporosis (GIO) research. The major advantage of mice in osteoporosis research, in contrast to rats or rabbits, is the possibility of testing the importance of genes in loss of functions, gain of functions and knock-in models. In common with humans, mice depict the strong decline of bone formation and some mouse strains also exhibit the early onset of resorption (reviewed in (Wood *et al*. 2017)). Most of the molecular mechanisms of GC action on the skeleton that were proven *in vivo* stems from these models. However, there are also certain limitations using mice. First of all, mice do not have osteons (Haversian system) in cortical bone, and thus, effects on cortical bone might differ from those in humans. Second, there is a strong variety in GC effects on bone concerning different mouse strains. Swiss Webster mice described by Weinstein and colleagues (Weinstein *et al*. 1998) are not suitable for studies of transgenic animals, since these are backcrossed into inbred strains, most often C57BL/6, 129SveV, Balb/c or FVB/N. C57BL/6 does not show strong alterations to GCs concerning their bone mass at younger age due to a relatively low bone mass. Unfortunately, most transgenic mouse strains are crossed to this background. Balb/c and FVB/N mice seem to be a better choice, since they show also alterations in bone mass at younger age due to GC (e.g. prednisolone treatment). For example, the studies with mice with conditional GR deletion or impaired GR dimerization were performed in FVB/N strains (Rauch *et al*. 2010). Arrival of CRISPR/Cas9-mediated gene editing, offers now the possibility to expand the functional tests of gene-encoding molecules in mice, and also in other species, which cover other aspects of human GIO. Finally, the application of GCs towards the animals to cause GIO varies greatly concerning ligands (dexamethasone, prednisolone and others), the route of application (intraperitoneal injections, slow-release pellets and oral gavage) as well as dosage and duration (from a couple of days until several months). This hampers the comparison of the different studies (reviewed in Wood *et al*. 2017). Here, standardization is of utmost need. Despite these shortcomings, much was learned from mouse models and the conclusions are detailed below.

### GC-induced osteoporosis

Long-term corticosteroid therapy leads to complex effects on bone. A rapid initial bone loss is followed by a slow constant long-lasting decline in bone mass (Canalis *et al*. 2007). In particular, this increases the fracture risk of the femoral neck and vertebrae (Weinstein 2012). The systemic actions of GCs, including the disturbance of Ca^2+^ absorption and reabsorption, sex-steroid levels and the growth hormone axis and increased muscle atrophy, may contribute to the adverse effects of GCs on bone. However, direct actions of GCs on bone cells seem to be more decisive in GIO development. These are the mesenchymal cell-derived bone-forming osteoblasts and their matrix-embedded descendants, the osteocytes, as well as the haematopoietic lineage-descended bone-resorbing osteoclasts.

**Figure 1:**
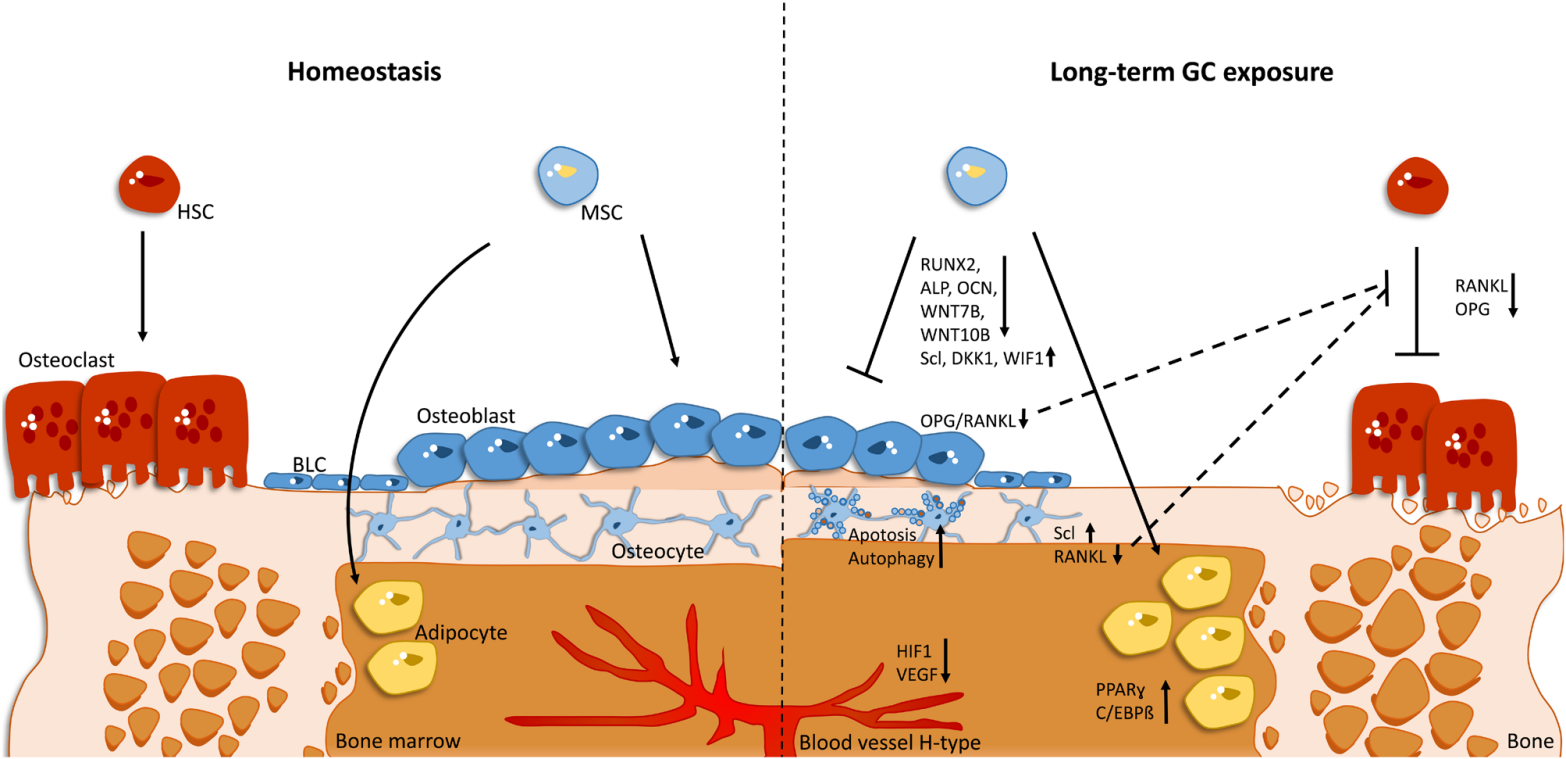
Effect of long-term glucocorticoid (GC) treatment on bone homeostasis. *Homeostasis:* In homeostasis (A), bone remodelling is balanced by the activity of bone-resorbing osteoclasts and bone-forming osteoblasts. The differentiation of osteoclasts from haematopoietic stem cells (HSC) is induced by binding of receptor activator of NF-κB ligand (RANKL) and is inhibited by osteoprotegerin (OPG). Osteoblasts derive from mesenchymal stem cells (MSC), which can also differentiate into fat-storing adipocytes. During bone formation, osteoblasts further differentiate into osteocytes or become bone-lining cells (BLC). H-type blood vessels provide nutrients and oxygen for bone cells. Long-term GC exposure: Long-term GC treatment reduces bone mass by a decreased osteogenic and concurrent increased adipogenic differentiation, leading to elevated bone marrow adiposity. This is caused by both a decreased expression of RUNX2, alkaline phosphatase (ALP), osteocalcin (OCN), and Wnt ligands (7b, 10b) and a simultaneous increase in expression of Wnt signalling inhibitors, including sclerostin (SCL), dickkopf-1 (DKK1), and Wnt-inhibitory factor (WIF1), as well as the adipogenic markers peroxisome proliferator-activated receptor γ (PPARγ) and CCAAT/enhancer-binding protein beta (C/EBP). Furthermore, osteoblasts and osteocytes synthesize less RANKL, and thus shift the RANKL/OPG balance towards less osteoclast differentiation and activity. In addition, osteoblasts and osteocytes undergo an increased amount of cell death (apoptosis) and autophagy. The supply of nutrients and oxygen by the specific H-type vascular subtype for bone cells is diminished by GC exposure via downregulation of hypoxia-inducible factor 1-alpha (HIF1) and vascular endothelial growth factor (VEGF). In summary, bone remodelling slows down on long-term GC exposure, leading to reduced bone mass.

Osteoblasts, osteocytes and osteoclasts work in concert in so-called bone-remodelling units to maintain bone mass. Bone resorption is initiated by the fusion of monocyte precursor cells to form osteoclasts in response to receptor activator of NF-κB ligand (RANKL) and other soluble and membrane-bound factors. In addition to osteoblasts, osteocytes were recently discovered to be a major source of osteoclast-inducing factors, including RANKL (Nakashima *et al*. 2011, Xiong *et al*. 2011) and others (Liu *et al*. 2017). The multinucleated osteoclasts generate a sealing zone at their basolateral side and create an acidic compartment that allows the degradation of mineralised matrix. Under normal physiological conditions, this process is terminated by a still poorly understood coupling mechanism that terminates osteoclast activity and subsequently activates osteoblast activity to form new bone followed by mineralisation (Henriksen *et al*. 2014) (Fig. 1).

The disturbance of these processes leads to bone loss because of enhanced bone resorption and/or diminished bone formation. Cell-type-specific genetic modulation of bone cells in mice confirmed the strong cell-autonomous impact of GCs on bone mass.

### GC excess on osteoclasts

Osteoclast activity is greatly increased at the onset of GC excess, but declines with prolonged GC excess. This dual activity results from complex, in part opposing, mechanisms of GCs on osteoclast function and maturation.

In particular, in cell systems, an induction of RANKL by simultaneously reducing the osteoclast differentiation inhibitor osteoprotegrin (OPG) was observed (Hofbauer *et al*. 1999, Rauner *et al*. 2011). However, in other studies, GCs failed to induce RANKL (Rauch *et al*. 2010, Piemontese *et al*. 2016). Intriguingly, RANKL inhibition by denosumab in humanized mice ameliorated to a certain extent the GC-mediated bone loss (Hofbauer *et al*. 2009). Genetic inactivation of RANKL in osteocytes reduced the cortical bone loss induced by GC treatment, but not in the trabecular bone. In conclusion, RANKL expression is at least in part involved in GC-mediated bone loss.

Intriguingly, osteoclast development from monocytic progenitors is suppressed by GCs (Jia *et al*. 2006, Kim *et al*. 2006), explaining the long-term decline of bone resorption during GC excess. This appears to be in part due to the impairment of the cytoskeletal reorganization influencing Rac activity and calpain 6 expression (Hong *et al*. 2011). However, increased osteoclast longevity was suggested to explain the initial enhanced resorption (Jia *et al*. 2006). Recently, it was found that the initial inhibitory effects of GCs on osteoclastogenesis are compensated by a direct increase of resorption activity of the remaining osteoclasts (Conaway *et al*. 2016, Shi *et al*. 2015) in a GR dimerization-dependent manner in mice (Conaway *et al*. 2016) , presumably by increasing reactive oxygen species (ROS) levels (Shi *et al*. 2015). Taken together, GCs act directly on osteoclasts, increasing their resorptive activity, and also lead to a decline in the long term of bone turnover (Fig. 1).

### GC excess on osteoblasts and osteocytes

Inhibition of bone formation is the major feature of GIO (Hartmann *et al*. 2016). This mainly results from impaired osteoblast function. Because osteocytes derive from the osteoblast lineage, GC impact on osteoblast abundance and function also contributes to the effect of GCs on osteocyte fate. Moreover, direct effects of GCs on osteocytes frequently lead to osteocyte death (O’Brien *et al*. 2004), which can be seen histologically as bone lacunas devoid of osteocytes, and thus, might have a direct impact on bone quality. We subsequently discuss here the impact of GCs on the development of the osteoblast lineage, on the proliferation, expansion and differentiation of osteoblasts and finally, on autophagy and apoptosis of osteoblasts and osteocytes.

### GC effects on progenitor cells and/or skeletal stem cells

The progenitor cells of osteoblasts are commonly considered to be mesenchymal stem cells (MSCs). These cells were originally defined as stromal bone marrow cells with multi- or at least tri-lineage differentiation potential into adipocytes, osteoblasts or chondrocytes. Finally, MSCs should be capable to give rise to new bone when transplanted ectopically into rodents (Caplan 1991). Numerous tissue-culture experiments demonstrated a decisive role for GCs in promoting MSC differentiation.

Some experimental conditions suggested that GCs contribute to adipocyte differentiation and that this would be at the expense of osteogenic differentiation, providing an attractive model to explain two side effects of GC excess, namely fat redistribution and GIO (Salem & Thiemermann 2010). Certain studies provide mechanistic clues that GCs are strong inducers of fat-cell-specific transcription factors, including C/EBPs, which subsequently induce peroxisome proliferator-activated receptor γ (PPARγ), the lineage-determining adipocytic transcription factor (Kawai *et al*. 2012). Accordingly, patients with GIO display high bone marrow adiposity (Rosen & Bouxsein 2006).

However, there are some conceptual problems with the cultures of so-called MSCs. For example, in GC-treated animals, bone marrow-derived MSCs display enhanced proliferation and subsequent mineralisation (Sui *et al*. 2016). A further major caveat is that the source for *in vitro* studies varied from bone marrow stromal cells to adipose-tissue stromal cells and ectodermal-derived calvarial pre-osteoblasts. Surprisingly, all of these cultures can be directed into distinct differentiation direction, given that the respective culture medium cocktail is provided. These cultures frequently contain cells of mixed character, and it remained unclear whether they originated from homogenous progenitors or whether these cultures consist of a mixture of distinct, specifically committed cells that expand only under the respective conditions. Furthermore, we observed that impaired GR dimerization in cells of GR^dim^ mice (described earlier) completely abrogates the induction of adipocytes from progenitor cells (Asada *et al*. 2011), but still suffices to mediate inhibition of osteoblastogenesis by GCs (Rauch *et al*. 2010). This points to the possibility that the molecular mechanisms of adipogenesis induction and osteogenesis repression by GCs are different.

Finally, the localisation of these MSCs *in vivo* was for a long time unclear. Recently, by lineage tracing, several mesenchymal and skeletal stem cells have been identified *in vivo* with multiple differentiation potentials, which are also activated during tissue repair (Ono *et al*. 2014, Chan *et al*. 2015, Worthley *et al*. 2015). These cells, defined by different transgenic mouse Cre lines, were able to give rise to multiple mesenchymal cell types, including osteoblasts, and are located close to the vasculature as perivascular cells (Kassem & Bianco 2015). Other osteoblast precursors are located at the periosteum (Grcevic *et al*. 2012). These periosteal progenitor cells have been identified by lineage tracing with Prx1-CreERT2, Osx-CreERT2, ɑSMACreERT2 and Gremlin1CreERT2 mice (Grcevic *et al*. 2012). These bone-lining cells can give rise to a substantial number of bone-forming osteoblasts when mature osteoblasts were genetically eliminated (Matic *et al*. 2016). GC treatment significantly reduced the bone-lining cells. However, whether the effects on bone-lining cells are pivotal in reducing bone formation currently remains unclear. To this end, also the bone marrow residing stem cells and their descendants need to be mapped under GIO conditions. In conclusion, whether GCs shift the balance of multipotent MSCs or only affect the differentiation of pre-committed osteoblast precursors, including bone-lining cells, currently remains to be determined.

### Osteoblast proliferation

Inhibition of osteoblast proliferation by GCs depends in part on direct regulation of cell cycle activators, for example, CDK2, 4, 6, CyclinD, c-Myc and E2F-1, and inhibitors, including p21 and p27, by the GR (reviewed in Komori 2016). Interference in MAP kinase pathways by the induction of dual specific phosphatase (DUSP)-1, a direct GR target, strongly ameliorates osteoblast proliferation (Horsch *et al*. 2007). However, DUSP-1 absence in knockout mice did not prevent GC-mediated bone loss, indicating a minor role for MAPK-pathway inhibition (Conradie *et al*. 2011).

### Osteoblast differentiation

One major mechanism of impaired bone formation is the inhibition of osteoblast differentiation. GR monomer activity as a molecular mechanism appears to be sufficient to suppress osteoblast differentiation *in vitro* and bone formation *in vivo*, because GR^dim^ mice display a normal response to GCs (Rauch *et al*. 2010). Pharmacological doses reduce the expression of key transcription factors for osteoblastogenesis, including runt-related transcription factor 2 (RUNX2) and osterix (OSX), the marker enzyme alkaline phosphatase, the late-stage marker osteocalcin and bone mineralisation (reviewed in Frenkel *et al*. 2015). Furthermore, Wnt ligands, which are decisive for osteoblast proliferation and differentiation, are reduced, in particular WNT7B and WNT10 (Mak *et al*. 2009). In contrast, Wnt inhibitors, including dickkopf-1 (DKK1), Wnt inhibiting factor 1 (WIF1) and sclerostin, are induced by GCs (reviewed in Hartmann *et al*. 2016).

Bone morphogenetic protein 2-dependent signalling strongly counteracts the negative effects of GCs on osteoblasts (Frenkel *et al*. 2015). GCs further act negatively on osteoblast differentiation by inducing Notch signalling (reviewed in Frenkel *et al*. 2015). Finally, inhibition of AP-1-dependent interleukin (IL)-11 expression by the monomeric GR appears to affect osteoblast differentiation substantially and explains the GR monomer-dependent bone loss (Rauch *et al*. 2010).

Recent studies suggested that in addition to GC-regulated proteins, microRNAs could also serve as effectors of GC actions. Although several microRNAs are described as important for osteoblast differentiation (reviewed in Hartmann *et al*. 2016), a recent study in mice with an osteoblast-specific deletion of Dicer, abrogating the generation of the majority of miRNAs, demonstrated that nonetheless the bone formation rate could still be diminished by GCs (Liu *et al*. 2016). Therefore, regulating miRNAs to inhibit bone formation is not essential for GC action on bone loss.

The reduction of osteoblasts and the accompanying decrease in bone formation was also attributed to GC-induced apoptosis. GC treatment increased the apoptosis rate of osteoblasts in mice and humans (Weinstein *et al*. 1998). This was prevented by bisphosphonates, calcitonin (Plotkin *et al*. 1999) and parathyroid hormone (PTH) administration (Jilka *et al*. 1999). Apoptosis in osteoblasts is initiated by the induction of the pro-apoptotic proteins Bim and Bak, in part via the induction of the transcription factor E4bp4 (Espina *et al*. 2008, Chang *et al*. 2009, Chen *et al*. 2014). Simultaneously, a decrease of the anti-apoptotic protein BcXL (reviewed in Komori 2016) occurs. GCs activate the kinase Pyk2 by Ca^2+^-influx induction, leading to Jun-N-terminal kinase (JNK) activation (Plotkin *et al*. 2007). By inducing an accumulation of ROS via activation of p66Shc kinase, GCs can further tip the balance towards cell death (Almeida *et al*. 2011).

To what extent osteoblast apoptosis contributes to bone loss still remains unclear. The fraction of apoptotic osteoblasts is elevated threefold by GC excess, but still does not represent a large percentage of the cells (Weinstein *et al*. 1998). Additionally, osteoblasts and osteoclasts from mice overexpressing 11β-HSD2 under the osteocalcin promoter to impair GC signalling in osteoblasts are resistant to elevated apoptosis (O’Brien *et al*. 2004). However, these mice display reduced bone mass. In contrast, vertebral compression strength is preserved in these mice. Therefore, while apoptosis contributes to GC actions on the skeleton, impaired bone formation appears to be less dependent on this process (O’Brien *et al*. 2004). In contrast to osteoblasts, osteocyte apoptosis is very high and particularly impacts on bone quality (Jilka *et al*. 2013).

### Effects of GCs on osteocytes

Osteocytes are embedded in bone matrix, residing in so-called lacuna. They are interconnected with dendritic processes and build with other osteocytes the lacuna-canalicular network (Dallas *et al*. 2013). As a major source of RANKL (Nakashima *et al*. 2011, Xiong *et al*. 2011) and because of to their expression of OPG and nitric oxide (NO), they modulate bone resorption. By releasing dentin matrix acidic phosphoprotein 1 (DMP1) and fibroblast growth factor 23 (FGF23), they also regulate phosphate homeostasis (Feng *et al*. 2006). Osteocytes on the one hand by expressing the Wnt inhibiting factors sclerostin (SOST, Scl), DKK1 and secreted frizzled-related protein (SRFP1) inhibit osteoblasts. On the other hand by increasing osteoblast-promoting NO and prostaglandin E2 osteocytes control bone formation (Dallas *et al*. 2013). *Sost* is upregulated during GC exposure in mice and rats (Sato *et al*. 2016, Beier *et al*. 2017), while *Sost* deficiency in part abrogates GC effects (Sato *et al*. 2016) by influencing crosstalk to resorption, but strikingly does not protect against GC-mediated inhibition of bone formation or stimulation of apoptosis. By contrast, Scl-neutralising antibodies prevent cancellous bone loss in part and apoptosis (Achiou *et al*. 2015), thereby restoring bone formation (Yao *et al*. 2016).

In addition to apoptosis induction, GCs were reported to induce macro-autophagy in osteoblasts, but also prominently in osteocytes. Intriguingly, lower GC dosage induced increasing autophagy in osteocytes rather than apoptosis (Jia *et al*. 2011), which is presumed to protect them from cell death. Using autophagy reporter mice (LC3-dsREd fusions), enhanced autophagy was detected *in vivo* upon prednisolone treatment (Yao *et al*. 2016). However, this was challenged by studies in mice lacking the autophagy key molecule Atg7 in late-stage osteoblasts and osteocytes (Piemontese *et al*. 2015). These mice exhibited less cancellous bone, but this was not further affected by prednisolone. Whereas prednisolone failed to increase autophagic flux in the mutant mice, there was no difference in cortical bone in response to prednisolone. The precise role of autophagy still needs to be carefully established, but presumably participates only partially in GC effects on bone.

### GC excess on vasculature

The vasculature has been recently established to have a decisive role in bone integrity and strength (Kusumbe *et al*. 2014). A specific vascular subtype, the so-called type H endothelium, is coupled to bone growth, builds a micro-environment for osteoprogenitor cells and is lost during ageing. Increasing hypoxia-inducible factor 1-alpha (HIF1) activity and subsequent vascular endothelial growth factor (VEGF) expression increases bone mass and osteoblast-marker gene expression in aged mice (Kusumbe *et al*. 2014). GC excess appears to affect HIF1 and VEGF expression, affecting vasculature volume and surface (Weinstein *et al*. 2010, 2017). GC signalling in osteoblasts in part appears to be decisive, because abrogation of GC signalling in OG2-11β-HSD2 transgenic mice prevents the decrease in vasculature volume.

### GC excess impairs growth

A striking clinical problem is the negative effects of GC excess on children’s growth by reducing bone growth. Withdrawal of pharmacological GC exposure leads to catch-up growth, an as yet very poorly understood phenomenon. Longitudinal bone growth depends on the proliferation and subsequent differentiation of chondrocytes in the growth plates. Chondrocytes differentiate to hypertrophic chondrocytes and the subsequent vascularisation of hypertrophic cartilage allows the replacement of cartilage with bone in the process of endochondral ossification. Overall, this process is controlled by the growth hormone (GH)–insulin-like growth factor 1 (IGF1) axis; whereas GH triggers IGF1secretion in the liver, both act on the cartilaginous growth plate in growing bones. GC excess is accompanied by a decline in GH and IGF1 titres as well as action (Jux *et al*. 1998), but this is challenged by studies reporting increased IGF1 receptor and GH receptor expression upon GC exposure (Heinrichs *et al*. 1994, Smink *et al*. 2002).

Exogenous GCs inhibit chondrocyte proliferation and increase hypertrophic chondrocyte apoptosis in the growth plate, resulting in impaired bone growth. Indeed, results from *ex vivo* cultures of human growth-plate cartilage suggest a differential regulation of Bcl-2 family member proteins by GCs, promoting apoptosis in proliferative chondrocytes (Zaman *et al*. 2014). By contrast, endogenous GCs appear to play a minor role in growth-plate chondrocytes, as indicated by the normal growth-plate phenotype of mice lacking the GR in these cells (Tu *et al*. 2014, and -reviewed in Hartmann *et al*. 2016).

### GC excess activating the mineralocorticoid receptor (MR)

The MR has a higher or similar affinity than the GR towards a variety of GCs such as the endogenous GCs cortisol and corticosterone, and still an affinity to the synthetic GR agonists beta-methasone and prednisolone (Lan *et al*. 1982). Thus, in part excess of endogenous GCs or high pharmacological concentrations of prednisolone also activate MR. Therefore, the GR/MR balance concept was proposed for tissues expressing both receptors, such as the brain (Joëls & de Kloet 2017). This led to a new concept: at low concentrations of GCs primarily MR is activated, whereas at high concentrations GR is activated and eventual both receptors exert distinct and overlapping function. Even the presence of GR/MR heterodimers is postulated. Tandem ChIP Seq analysis showed binding regions for both receptors, suggesting them in a common complex in the hippocampus (Mifsud & Reul 2016). MR and GR expression was determined in human foetal bones by Beavan and colleagues (Beavan *et al*. 2001) and indicated immunoreactivity and expression for both receptors in osteoclasts, osteoblasts, and osteocytes. Therefore, for bone the MR/GR balance concept could be valid as well. The functional involvement of MR was addressed by Ikeda and colleagues who blocked MR function with eplerenone (Fumoto *et al*. 2014). In a model of GIO by application of prednisolone this pharmacological blockade of MR lead to a decreased reduction of trabecular and cortical bone mass. Unfortunately, whether inhibition of MR leads to an attenuated reduction of bone formation rate by prednisolone was not analysed. Interestingly, the lack of MR in late-stage osteoblasts and osteocytes using MRflox;Dmp1Cre mice showed less reduction of trabecular bone in femur by GCs than control mice, whereas the reduction of cortical thickness was completely unaffected (Fumoto *et al*. 2014). Taken together, MR contributes to GC excess mediated bone loss eventually in part by acting on osteocytes, however, the role of MR in individual bone cell types seems to be less important than the GR.

### Effects of selective GR ligands on bone

The discrimination of GR monomer function and GR dimerization was also addressed pharmacologically since the 1990s. The aim was to favour transrepression of pro-inflammatory genes by the GR monomer and to avoid the transactivation of metabolic acting genes by GR dimers. The hope was to identify GR ligands with anti-inflammatory efficacy similar like classical GR agonists, but with a better side effect profile, including sparing of the bone (Schäcke *et al*. 2007). Given the findings in mice with impaired GR dimerization and what we know nowadays about the molecular requirements to suppress inflammation, this concept was retrospectively too simplistic, but state of the art in those days (Beato *et al*. 1995). The first generation were so-called selective GR agonists (SEGRAs) that still exert a steroid backbone and bind to the ligand pocket of the GR (Schäcke *et al*. 2007). These compounds possessed anti-inflammatory efficacies in certain distinct inflammatory paradigms, such as croton-oil induced inflammation. Of note for croton-oil induced inflammation was one of the few inflammatory diseases, in which also GR^dim^ mice (with intact GR monomer) turned out to be responsive (Reichardt *et al*. 2001). In contrast GR^dim^ mice failed for most other inflammatory diseases tested so far to exert reduction of inflammation by classical GR agonists. Accordingly, compounds for topical treatment of skin inflammation, such as mapracorat (ZK245186), are now tested in clinical trials for atopic skin diseases. However, some of these compounds with steroidal scaffold still exert side effects (reviewed in Strehl *et al*. 2017). The next generation compounds were so-called selective GR modulators, SEGRMs with non-steroidal scaffolds (reviewed in Sundahl *et al*. 2015). These modulators are not binding to the ligand-binding pocket. The most prominent example is the shrub-derived compound A (CpdA). Recently, for SEGRAs, the anti-inflammatory efficacy was increased by introducing electrophilic covalent-binding warheads to improve longer residence of the ligands at the GR binding pocket (Chirumamilla *et al*. 2017). Because of the overall findings that GR dimerization is crucial to confer suppression of inflammatory processes via transactivation of anti-inflammatory acting genes (such as *Dusp1, Anxa1, Sphk1* and others), also GR dimer inducing agents were postulated to improve steroid therapy (De Bosscher *et al*. 2016). Apart from the concept of GR dimer/monomerization selectivity, ligands were developed that induce a differential shift of the helix 12 in the ligand-binding domain. Accordingly, this leads to different co-activator/co-repressor interaction. Indeed such compounds also might exert a decreased side effect profile (Hu *et al*. 2011).

Comprehensive information on how these selective GR agonists/modulators affect bone is currently lacking. Humphrey and colleagues analysed the effect of several compounds (RU24858, RU40066, RU24782, AL438-F1 and ZK216348) on the OPG/RANKL ratio. They concluded that these compounds suppress the OPG/RANKL ratio less than prednisolone in two human osteoblast cell lines (Humphrey *et al*. 2006), suggesting that bone resorption by osteoclast is less induced than with full GR agonists. For the GR modulator PF-802, exerting different Helix-12 shifts and thus co-regulator recruitments, a lack of suppression of osteocalcin was described (Hu *et al*. 2011) *in vitro*.

Some of the selective agents were, however, analysed in more detail on bone metabolism *in vivo*. For the GR modulator AL-438 selected on the base of differential co-regulator recruitment, a clear lower suppression of bone formation rate was described, thus sparing GC effects on bone (Coghlan *et al*. 2003).

Another striking compound leading to differential co-regulator recruitment is LGD-5552. LGD-5552 does not impair bone formation rate at lower concentrations but still exert anti-inflammatory effects (Miner *et al*. 2007).

The GR mimetics containing diazaindole moieties ((R)-18 and (R)-21 were described to have less effect on bone mass than prednisolone. And this was demonstrated even at concentrations showing a stronger reduction of inflammatory disease score than prednisolone in a model of collagen-induced arthritis (Harcken *et al*. 2014). This was one of the few studies that examined GIO in the context of inflammation with novel GR modulators.

The shrub derived CpdA, a non-steroidal GR modulator showed initial promising results by not inducing RANKL/OPG ratio (Rauner *et al*. 2011), and not affecting osteoblast differentiation (Rauch *et al*. 2011). Furthermore, CpdA was suppressing IL11 expression in osteoblasts (Rauch *et al*. 2011). Since IL-11 is suppressed in mice with impaired GR dimerization and is decisive for inhibition of osteoblast differentiation (Rauch *et al*. 2010), this leads to the assumption that CpdA might not induce GR-AP-1 interference in stromal cells (De Bosscher *et al*. 2013). *In vivo*, CpdA indeed did not affect bone formation and was not increasing resorption in mice (Thiele *et al*. 2012). Despite these positive findings, the toxicity of CpdA due to rapid degradation into toxic products dampens the promise of this compound. However, these results show that tissue selective GR modulation as exerted by CpdA (Rauch *et al*. 2011, Thiele *et al*. 2012) or (R)-18 and (R)-21 (Harcken *et al*. 2014), could be a strategy to combat certain inflammatory diseases and spare bone mass.

### Endogenous GC action on bone

Endogenous GCs are necessary for physiological bone metabolism. Targeted overexpression of 11β-HSD2 in osteoblasts and osteocytes in Col2.3-11 β-HSD2 transgenic mice led to reduced cortical and trabecular bone mass (Sher *et al*. 2004, 2006, Kalak *et al*. 2009), but only at selected sites, and delayed cranial-bone development (Zhou *et al*. 2009). Augmented Wnt signalling by low dose GCs, but shut down by high-dose GCs, could be one mechanism of anabolic effects of GCs at physiological concentrations (Mak *et al*. 2009). By contrast, studies conducted in aged mice demonstrated increased skeletal fragility by endogenous GCs, leading to impaired bone angiogenesis and decreased vasculature volume (Weinstein *et al*. 2010).

The effects of endogenous GCs could be exerted by both MR and GR. Due to the higher affinity of MR towards GCs, effects of low GC concentration could be rather mediated by the MR than the GR. However, ablation of the MR in osteoblasts in MRflox;OsxCre mice and in osteocytes in MRflox;Dmp1Cre mice did not alter trabecular bone mass substantially (Fumoto *et al*. 2014). Nonetheless, blockade of wild type mice with the MR antagonist epleronone lead to an increase of trabecular bone mass, bone formation rate, and reduced osteoclasts. Thus, the MR impacts negatively on basal bone integrity (Fumoto *et al*. 2014). However, the cell types involved remain to be elusive, e.g. the role of MR in osteoclasts, but also in complete other organs can be anticipated. It also remains unclear whether the MR mediates GC effects at all, since ablation of GC signalling rather decreases bone mass. A negative impact of aldosterone acting via the MR can not be excluded.

In contrast to blockage of MR, but similarly to inhibition of GC signalling (overexpression of 11β-HSD2 and lack of 11β-HSD1), the deletion of GR in osteoblasts leads to reduced bone mass. Ablation of GR in osteoblasts decreases bone volume in the trabecular compartments of particular bones when using GR^Runx2Cre^ mice (Rauch *et al*. 2010). Postnatal tamoxifen-induced ubiquitous deletion of the GR in GR^gtROSACreERT2^ mice resulted in reduced cancellous bone only in the tibia, and not in the femur or vertebrae (Rapp *et al*. 2018).

Taken together, dependent on the model, GCs and the GR are slightly anabolic at very distinct bone sites, either femora, vertebrae or tibia. In contrast, MR seems to be rather catabolic and whether it mediates GC effects remains to be elucidated. The different background strains of the respective mouse lines may also contribute to the differential effects on bone mass.

## Role of GCs on bone fracture healing

### Clinical problems

Patients with GIO are at increased risk for fractures, and it can be anticipated that the process of fracture healing is disturbed because of the strong effects of GCs on virtually all participating cell types. However, there are no clinical studies on fracture healing in GIO patients. Furthermore, to date clinical data on bone healing in patients under short-term GC medication are lacking. However, such observations would be extremely important, because GCs are widely prescribed for patients with inflammatory and other disorders. In the following section, we review the literature on the effect of exogenous and endogenous GCs on fracture healing in preclinical models.

### Fracture-healing process and participating cell types

Fracture healing starts with a fine-tuned inflammatory phase with the orchestrated actions of immune cells and cytokines. Because of tissue and vessel disruption, endogenous danger associated molecular patterns (DAMPs), including histones, mitochondrial DNA, and ATP, are released, which trigger activation of the immune system (Schaefer 2014). Immediately, the coagulation cascade is activated, leading to haematoma formation at the fracture site, which is characterised by high lactate, low pH, hypoxia, and immune cells from the blood (Kolar *et al*. 2011, Hoff *et al*. 2016). Because of DAMP activity and activation of the coagulation system, the complement cascade is activated, whereby, among other complement molecules, the anaphylatoxins C3a and C5a are produced (Ehrnthaller *et al*. 2011). C5a in combination with inflammatory mediators released by cells resident in the fracture area and adjacent bone marrow, for example, macrophages, mast cells, mesenchymal cells and endothelial cells, lead to the recruitment of immune cells. The first cells recruited in a high number to the damaged area are polymorphonuclear neutrophils, which secrete chemo-attractive cytokines to attract other cells, including monocytes and macrophages, which further clear the fracture zone. In addition to cells of the innate immune system, B- and T-lymphocytes invade the fracture zone and further modulate the inflammatory milieu. When the inflammatory reaction subsides, the healing cascade progresses to the regenerative phase. Here, periosteal progenitor cells start to proliferate and new bone is deposited via intramembranous ossification remotely from the fracture. Near the fracture, mesenchymal progenitor cells differentiate into the chondrocyte lineage and follow the path of endochondral ossification. Once the fracture gap is bridged by cartilage and thus the mechanical environment is more stable, the callus progressively becomes vascularized and ossification advances from both sides towards the fracture until all cartilage is replaced by woven bone. Subsequently, the callus is resorbed by osteoclasts, remodelled to lamellar bone, and the original bone shape re-established.

Proper resolution of inflammation is mandatory to enable normal fracture healing. Any disturbance of the inflammatory phase is known to influence fracture healing outcome negatively; examples are prolonged inflammation, increased or altered profile of inflammatory mediators as probably caused by concomitant inflammatory disorders, including severe trauma, rheumatoid arthritis, osteoporosis, and diabetes, and experimental depletion of certain immune cells (Mountziaris *et al*. 2011, Claes *et al*. 2012, Einhorn & Gerstenfeld 2015).

GCs could participate in the resolution of inflammation also during fracture healing. However, they have both adverse and beneficial roles on bone cells, depending on the exposure and timing. However, how GCs orchestrate fracture healing remains largely unknown and only recent studies with conditional-knockout mice shed some light into this complicated process, as described below.

### GC excess during fracture healing

Interestingly, only a few studies to date have analysed the effects of GCs on fracture healing. These studies demonstrated different effects of short- and long-term administration of synthetic GCs on bone repair. Short-term medication with GCs did not significantly disturb bone regeneration (Aslan *et al*. 2005), whereas long-term administration initiated prior to fracture impaired bone regeneration (Waters *et al*. 2000). Radiographically, significantly fewer unions were detected together with reduced bone mineralisation, indicating delayed healing (Waters *et al*. 2000). In agreement with these findings, biomechanical properties of femoral-shaft fractures were significantly reduced after GC administration (Sandberg & Aspenberg 2015). The reasons for the delayed bone regeneration under long-term GC treatment are not fully understood. In a mandibular-defect model, impaired osteogenic differentiation, indicated by reduced staining for RUNX2 and osteocalcin, was reported after short-term dexamethasone administration. However, at the endpoint, no differences between control and dexamethasone treatment were noted, indicating a transient effect (Li *et al*. 2012). Earlier studies reported suppression of collagen synthesis by dexamethasone in calvarial cells (Lukert *et al*. 1991, Advani *et al*. 1997), but mechanistic studies on bone regeneration are, to the best of our knowledge, lacking.

### Endogenous GCs during fracture healing

Only a few very recent studies addressed the role of endogenous GCs by studying mice with impaired GC signalling (Weber *et al*. 2010) or GR deletion (Tu *et al*. 2014, Rapp *et al*. 2018). Using Col2.3-11β-HSD2 mice, disrupting GC signalling in osteoblasts did not affect intramembranous bone healing induced by drill-hole defects in the proximal tibia (Weber *et al*. 2010). By contrast, GR deletion in chondrocytes in GR^Col2CreERT2^ mice attenuated endochondral bone healing in tibial metaphyseal fractures by transiently increasing the cartilaginous fraction of the callus, but still resulted in a normal healing response (Tu *et al*. 2014).

**Figure 2:**
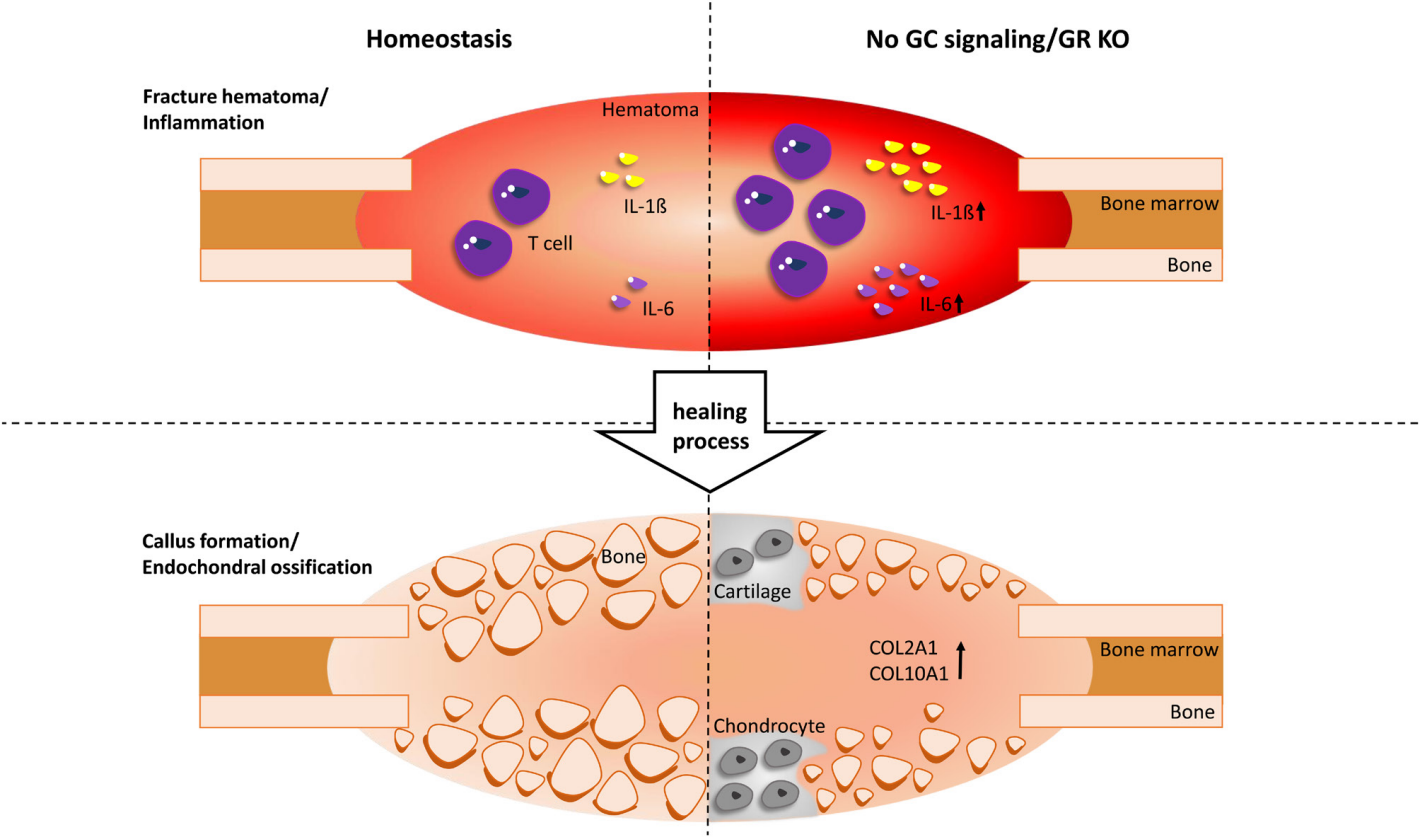
Effect of GCs on fracture healing. During the *inflammation phase*, in which a fracture haematoma is created, the absence of the glucocorticoid receptor (GR KO) causes an increased inflammatory response. This is shown by elevated interleukin (IL)-6 and IL-1ß levels as well as increased T-cell infiltration. During *callus formation*, endochondral ossification by chondrocytes is disturbed by persisting cartilage, as confirmed by an elevated expression of collagen type 2 (COL2A1) and collagen type 10 (COL10A1) and later, bony bridging of the fracture gap is reduced. In summary, the GR has a protective role in fracture healing by influencing the inflammatory response and by promoting cartilage-to-bone transition.

Because the fracture-healing process requires many different cell populations, we recently investigated mice with a tamoxifen-inducible deletion of the GR, GR^gtROSACreERT2^ mice, in all cells, including bone and immune cells (Rapp *et al*. 2018). These mice were subjected to a femur osteotomy that was stabilized using an external fixator to allow a reproducible endochondral healing response. The absence of the GR in the immune system in GR^gtROSACreERT2^ mice caused a greater inflammatory response at the fracture onset, confirmed by elevated interleukin (IL)-6 levels in the serum, increased IL-1β concentrations in the initial fracture haematoma and a significantly higher number of T cells infiltrating the fracture callus. During callus formation, endochondral ossification was disturbed in the absence of the GR, as confirmed by persisting cartilage in GR^Col2CreERT2^ mice. In the late healing phase, bony bridging of the fracture gap was reduced, resulting in poor mechanical properties of the healed bones. Therefore, the GR has a protective role in fracture healing by shaping the inflammatory response and by promoting cartilage-to-bone transition (Tu *et al*. 2014, Rapp *et al*. 2017) (Fig. 2).

## Bone regeneration and GCs

Because GCs affect fracture healing and regulate proliferation/apoptosis and differentiation of bone precursor and mature bone cells, GCs are also involved in bone regeneration in non-mammalian vertebrates, albeit little is known about their action in this process.

While only some organs regenerate well in adult mammals, non-mammalian vertebrates, in particular salamanders and some teleost fish species, efficiently and completely restore complex organs and structures that do not regenerate in mammals, including appendages (limbs, fins and tails). Bone represents a major tissue in appendages; intriguingly it is completely regenerated after amputation of salamander limbs and fish fins. As discussed previously in this review, progenitor cells are thought to be the cellular source of newly forming osteoblasts during mammalian bone repair, whereas mature osteoblasts (osteocalcin positive) do not appear to contribute to bone repair (Park *et al*. 2012, Chan *et al*. 2015, Worthley *et al*. 2015). Interestingly, non-mammalian vertebrates appear to employ additional cellular mechanisms for bone repair and regeneration. While the source of regenerating osteoblasts in salamander limbs is not yet known, in zebrafish fins differentiated *osteocalcin*-positive osteoblasts dedifferentiate in response to amputation, revert to a progenitor status and provide a source of regenerating osteoblasts (Knopf *et al*. 2011).

Very little is known about the molecular mechanisms controlling osteoblast dedifferentiation. The only pathway that has been implicated to date is retinoic acid signalling, which must be downregulated for osteoblast dedifferentiation to occur (Blum & Begemann 2015, Sehring *et al*. 2016). To determine additional regulators of this intriguing process, we have employed an unbiased small-molecule screen, which revealed that treatment with exogenous GCs can enhance dedifferentiation (R Mira and G Weidinger, unpublished observations). It will be very interesting to characterise the role of endogenous GC signalling in osteoblast dedifferentiation.

While this is ongoing work, the influence of GCs on other aspects of bone biology in non-mammalian species has begun to attract the attention of several researchers. Zebrafish in particular are an attractive model system for *in vivo* studies of bone formation, repair and regeneration, which are also amenable to large-scale approaches, which could be instrumental for the identification of therapeutically relevant modifiers of bone repair. The negative effects of high-dose GC treatment on bone are conserved in zebrafish, indeed, models of GIO have been established in larvae and in adult scales (Barrett *et al*. 2006, de Vrieze *et al*. 2014, Pasqualetti *et al*. 2015). GCs can also inhibit regenerative growth of zebrafish fins after amputation, as revealed in small-molecule screens for modifiers of regeneration (Mathew *et al*. 2007, Oppedal & Goldsmith 2010). As in mammals, systemic GC treatment of zebrafish has complex effects, including suppression of the immune response, reduced osteoblast differentiation, and proliferation, and effects on osteoclast activity (Geurtzen *et al*. 2017). Tissue-specific manipulations of GR activity have not yet been performed in zebrafish, thus it has to date not been possible to ascertain which of the GC effects on bone, and in particular on osteoblasts, are direct. Interestingly however, in contrast to mammals, prednisolone treatment does not appear to induce osteoblast apoptosis in regenerating zebrafish fins (Geurtzen *et al*. 2017). The metabolism of endogenous GCs in zebrafish could further differ from mammalians due to the lack of 11β-HSD1 expression (Baker 2004). Further studies will be needed to test whether other aspects of GC action on bone also differ between mammals and highly regenerative non-mammalian species, and to what extent regeneration-specific events are regulated by endogenous GR signalling.

## Conclusion

GCs and their receptor, the GR, substantially affect the skeleton. Effects of GCs have been intensively studied over recent decades in model organisms, and clinical data about the detrimental effects are abundantly available. Using mouse models with selective ablations of GC signalling and the GR provided some mechanistic knowledge about the requirement of the GR in chondrocytes, osteoblasts and osteoclasts as well as intriguing insights into the cell-autonomous role of the GR concerning proliferation, apoptosis and differentiation of bone cells under steady-state conditions and pharmacological GC exposure. The analysis of the chromatin landscape coupled to GR transcriptional activity – despite being exploited in metabolic tissues and immune cells – has to date not been performed regarding the action in skeletal cells. Single-cell sequencing in well-designed experiments will help to dissect the still ill-understood mechanisms of different GC exposure on skeletal cells that can be anabolic or catabolic. Furthermore, we have just started to understand how the GR affects the crosstalk between different skeletal compartments (e.g. vasculature and bone-lineage cells, immune system and the skeleton). Advances in *in vivo* imaging will help to unravel the effects of endogenous and exogenous GCs on the complex processes of bone growth, remodelling and regeneration and fracture healing. This basic research is of utmost importance to understand the pathophysiology of aberrant GC signalling as it occurs during steroid therapies, stress, chronic inflammation and ageing. Only then can we provide proper rationales for therapeutic concepts that allow either time-dependent or tissue-specific delivery of GCs, the development of GR modulators addressing distinct molecular mechanisms to improve therapeutic efficacy and to develop strategies to specifically target GC's adverse effects on bone.

## Declaration of interest

The authors declare that there is no conflict of interest that could be perceived as prejudicing the impartiality of this review.

## Funding

This work was funded by the German Research Foundation within the Collaborative Research Centre ‘Danger Response, Disturbance Factors, and Regenerative Potential after Acute Trauma’ (CRC 1149, Subproject bib2 and bib3 to JT, AI and GW).
